# Evidence of vascular involvement in myopia: a review

**DOI:** 10.3389/fmed.2023.1112996

**Published:** 2023-05-18

**Authors:** Alexandra Benavente-Perez

**Affiliations:** Department of Biological Sciences, State University of New York College of Optometry, New York, NY, United States

**Keywords:** myopia, vascular, structure, function, evidence

## Abstract

The benign public perception of myopia (nearsightedness) as a visual inconvenience masks the severity of its sight-threatening consequences. Myopia is a significant risk factor for posterior pole conditions such as maculopathy, choroidal neovascularization and glaucoma, all of which have a vascular component. These associations strongly suggest that myopic eyes might experience vascular alterations prior to the development of complications. Myopic eyes are out of focus because they are larger in size, which in turn affects their overall structure and function, including those of the vascular beds. By reviewing the vascular changes that characterize myopia, this review aims to provide an understanding of the gross, cellular and molecular alterations identified at the structural and functional levels with the goal to provide an understanding of the latest evidence in the field of experimental and clinical myopia vascular research. From the evidence presented, we hypothesize that the interaction between excessive myopic eye growth and vascular alterations are tipping-points for the development of sight-threatening changes.

## 1. Introduction

The blinding consequences of myopia are often overlooked (1–7). Myopic eyes are larger in size, which alters not only their focusing ability, but also their anatomy and physiology (8). All myopes, regardless of degree, are at increased risk of visual impairment (3, 9, 10). This has significant implications due to the predicted global increase in myopia prevalence and the potential public health crisis it represents (8, 11). As we learn about the role that peripheral refraction (12, 13), light intensity (14, 15), time spent outdoors (16, 17), or the on/off pathways play in the development of myopia (18, 19), the controversy is no longer whether myopia is genetic or environmental, but whether we can identify the variables that interact in this multifactorial condition. Currently, there are no preventive markers for myopic degeneration, which is predicted to threaten the eyesight of five billion people by 2050. Myopic eyes have thinner choroids and scleras and, if they progress into high myopia, they can have secondary macular defects in Bruch’s membrane along with a complete loss of retinal pigment epithelium, choriocapillaris, and retinal photoreceptors, which confirms the effect of myopia on the ocular vasculature.

The eye’s vascular network comprises a complex grid of supply and drainage structures. The retina has a high metabolic rate and oxygen consumption per unit weight in the body (20). In humans, the retina is supplied by the central retinal artery (CRA) - directly in charge of the inner two thirds by diffusion to rods, cones and outer layers - and the choroid, supplying the outer third. The retina is particularly vulnerable to ischemia because of its high oxygen demand and low vascularity of the inner layers (21, 22). The choroid has a high flow rate, low oxygen exchange and a fenestrated capillary bed. While the choroidal circulation is mainly controlled by sympathetic innervation and thought not to be autoregulated (23), the retinal circulation is ruled by locally controlled autoregulatory mechanisms, including mediators released by endothelial cells (23). A web-like capillary network spreads throughout the retina to provide additional supply (24), connects arteries and veins, and allows direct transport of oxygen, water and lipids to the tissues by diffusion. Capillaries are most abundant in the macula but absent from the fovea (capillary-free zone), which obtains its nutrients from the choriocapillaris (25). The superficial optic nerve head zone is supplied by the central retinal artery, while the short posterior ciliary arteries supply the lamina cribrosa (24, 26).

Until recently, the perfusion features of the human myopic eye had only been studied in human pathological myopia (27, 28) and experimental models of myopia (29–33). This review aims to summarize the latest evidence and controversies in the field of experimental and clinical myopia vascular research by addressing the structural and functional gross, cellular and molecular vascular alterations identified in myopic eyes.

## 2. Vascular features of the human myopic eye

Most of the techniques used to assess ocular hemodynamics *in vivo* are non-invasive imaging systems that assess retinal blood-flow velocity directly or indirectly (laser doppler velocimetry, LDV) (34), oxygen saturation (oximetry) (35), capillary perfusion (optical coherence tomography angiography, OCTA) (36), microvascular health (adaptive optics scanning light ophthalmoscope fluorescein angiography, AOSLO FA) (37), blood flow (laser speckle contrast imaging LSCI) (38), choroidal pulsatile ocular blood flow (POBF) (39), retrobulbar blood velocity (color doppler imaging, CDI) and retinal blood velocity (laser doppler velocimetry, LDV) (34), amongst others. Until recently, the only method providing a measure of absolute retinal blood flow was the combination of LDV blood velocity with retinal vessel diameter measures from fundus photographs (40, 41). This technique is time-consuming and impractical in the clinic. The recent development of doppler optical coherence tomography (DOCT) provides full quantitative volumetric information of blood flow and vascular/structural anatomy (42). Due to the variety of techniques available, it is imperative to consider the unique technical, anatomical and clinical characteristics of each instrument when interpreting outcomes.

**Figure 1:**
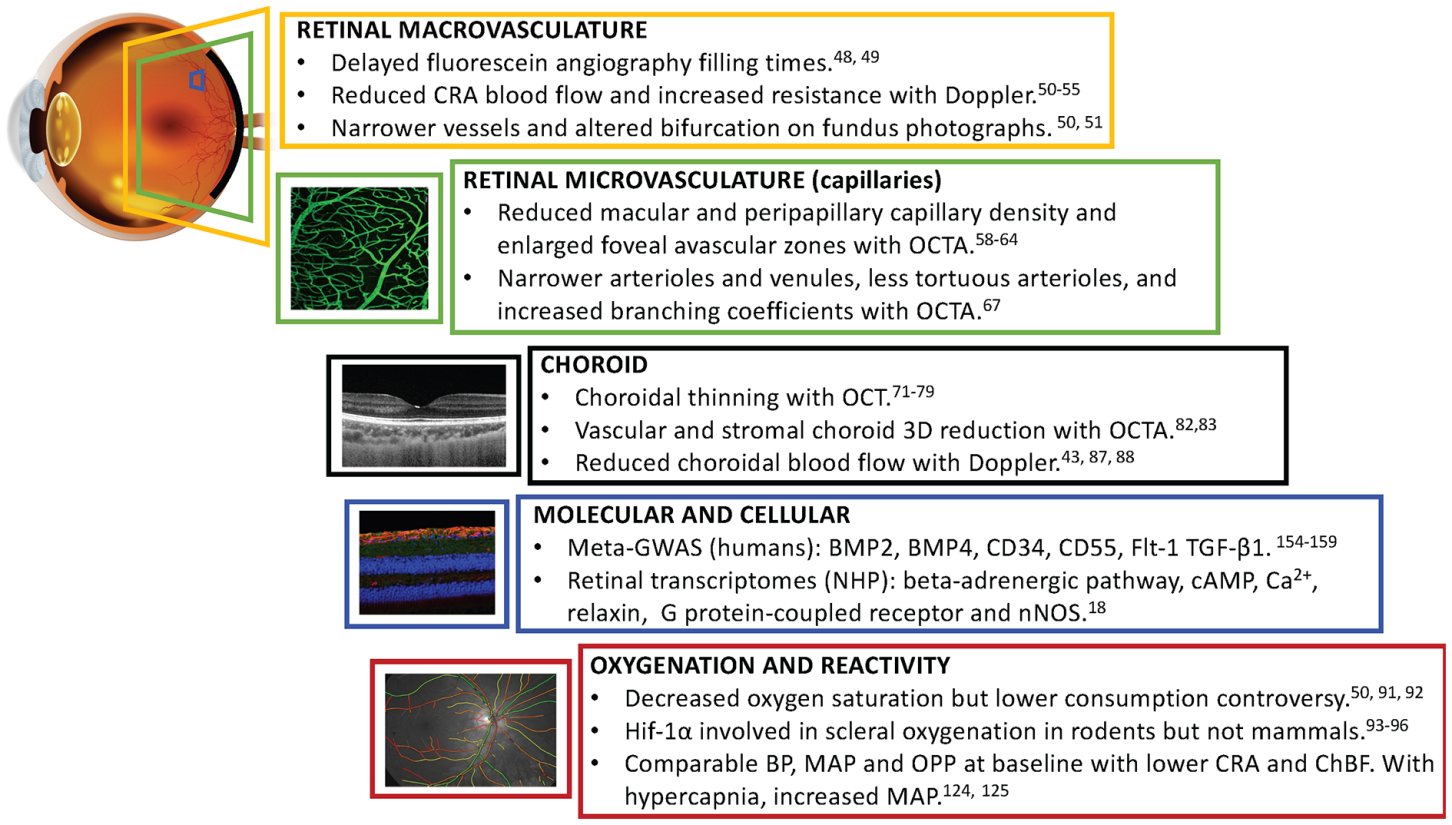
Schematic summary describing key vascular findings identified in myopic eyes to date. CRA, central retinal artery; OCTA, optical coherence tomography angiography; BMP2 and BMP4, bone morphogenetic proteins 2 and 4; CD34 and CD55, transmembrane proteins CD34 and CD55; Flt-1, vascular endothelial growth factor receptor-1; TGF-β1, transforming growth factor beta receptor; cAMP, cyclic adenosine monophosphate; Ca^2+^, calcium; nNOS, neuronal isoform of nitric oxide; Hif-1α, hypoxia-inducible factor 1-alpha; BP, blood pressure; MAP, mean arterial pressure; OPP, ocular perfusion pressure; ChBF, choroidal blood flow.

It is hypothesized that the compromised hemodynamics observed in young healthy myopes is an early feature of the decreased ocular blood flow reported in pathological myopia. Such vascular features would increase the susceptibility of the myopic eye for vascular and age-related eye diseases. For instance, impaired retinal blood flow might increase the risk to develop chorioretinal atrophy in high myopia. These changes possibly interact with the known effect of aging on the retinal and choroidal vasculature, including decreased tissue perfusion, deep capillary plexus vessel density, venous, capillary and choroidal blood flow and loss of endothelial cells amongst others (43–50). Below we review experimental and clinical evidence suggesting the existence of vascular alterations in physiological and degenerative myopia (Figure 1).

## 3. The effect of myopia on the retinal vasculature

The retinal vasculature provides metabolic support to neural and glial cells while minimally interfering with light-sensing mechanisms (51). One of the first pieces of evidence describing an altered retinal circulation in myopic eyes was the discovery of delayed filling times in the arterial, arterial–venous and venous phases of high myopic eyes using fluorescein angiography in the 1970s (52, 53). Subsequently, myopic eyes have been found to exhibit narrower vessel diameters (54), altered bifurcation (55) and reduced central retinal artery (CRA) diameter and blood flow (56–59). This effect appears localized to the CRA once it branches out of the ophthalmic artery (OA), and therefore affects the inner retina but not structures supplied by the OA such as eyelids, lacrimal gland, conjunctiva, posterior uveal tract or extraocular muscles. In degenerative myopia, both choroidal and retinal blood flow appear reduced, which has been hypothesized to be partly due to increased vascular resistance or adaptive changes the myopic eye experiences to cope with its enlargement (27, 28, 60).

At the retinal microvascular level, healthy myopic eyes show reduced macular superficial, deep and radial peripapillary capillary vessel density, along with increased density inside the disc and enlarged foveal avascular zones (61–67). There is controversy, however, to whether these reductions in capillary densities actually result in altered capillary blood flow (63, 64), since there is evidence that reductions in capillary density are not necessarily associated with choroidal thickness (66) or retinal nerve fiber layer function (68). In terms of anatomy, myopic eyes with greater axial lengths exhibit narrower and less tortuous arterioles and venules, and greater branching coefficients (69). The lower capillary density observed in myopic eyes has been proposed to be a protective mechanism for decreased risk of diabetic retinopathy, but the protective effect has not been confirmed in later studies (70).

The alterations observed in healthy myopic eyes may be precursors of changes seen in pathological myopia and possibly involved in the pathophysiology of myopic degeneration: decreased density of the deep radial capillary plexus and a reduction in OA blood flow that relates to the severity of the retinal degeneration (27).

## 4. The effect of myopia on the choroidal vasculature

Choroidal thickness is a marker of myopia development first identified in experimental myopia in avian eyes (32, 71). In fact, gross anatomical changes in choroidal appearance from myopic chick eyes led to a series of publications confirming bidirectional choroidal thickness changes in response to defocus (72). These changes have been observed in mammal, non-human and human primate eyes (72–80). In addition, during childhood the choroid thickens with normal eye development, but to a lesser extent in children developing myopia, which confirms the role the choroid may play during myopia development (81, 82). There is also evidence of a three-dimensional reduction in choroidal vascular and stromal components in myopic eyes, mainly in the nasal and subfoveal region (83), although this vascular thinning remains controversial (84). The ocular pulsatile blood flow, thought to be mainly choroidal (85), also appears reduced in myopic eyes (39). This reduction, however, might be an artefact of enlarged eye volume (56, 86), since ocular pulsatile calculations depend on intraocular volume (87). In experimental models of myopia, choroidal flow reductions relate to thickness changes, suggesting an altered choroidal supply and thickness changes that might be responsible for choroidal flow changes or vice versa (88). However, this relationship has not been confirmed in humans - choroidal blood flow might remain constant in eyes with non-pathological myopia (89). There is also evidence that moderate, but not high myopes, exhibit greater ocular perfusion pressure as the choroid thins (90), and choriocapillaris flow deficits are greater in high myopes with no pathology (91). This suggests that early vascular anatomical and functional differences between moderate and high myopes need to be evaluated longitudinally and might represent clinically applicable biomarkers of early pathologic myopia.

A major cause of visual impairment in pathological myopia is loss of photoreceptors, which are nourished by the choroid. Therefore, any choroidal dysfunction can have detrimental consequences in myopic eyes. In fact, eyes with degenerative myopia exhibit lower posterior ciliary artery blood flow, which supply the choroid (27). Choroidal structure measures such as thickness, luminal and stromal area, and choroidal vascularity index are also significantly reduced in pathological myopia. In addition, thickness and vascularity index appear associated with degeneration severity and visual acuity, highlighting the role the choroid represents for degenerative myopia (92, 93).

## 5. Retinal oxygen saturation in myopia

The retina is characterized by its high metabolic rate and considered one of the tissues with the largest oxygen consumption per unit weight in the body (20). Retinal oximetry performed using commercially available systems like the Oxymap has identified a lower arterio/venous oxygen saturation ratio in myopic eyes that points towards a possibly lower retinal oxygen consumption (94). However, a significantly larger cross-sectional study with 1,461 participants found that when age, gender, body mass index (BMI), intraocular pressure (IOP) and axial length (AL) were corrected for, longer and more myopic eyes did not exhibited a lower, but a greater oxygen and arterio-venous ratio saturation (95). These findings suggest that as eyes grow larger, they might be able to maintain an adequate oxygenation profile for its growing size, until they reach a degenerative state and the oxygenation profile is affected (96). There is also work suggesting that myopic choroidal thinning and reduced choroidal blood flow might affect scleral oxygenation (97). If this is correct, manipulating hypoxia signaling pathways might be a myopia control alternative in the future (98). Recent evidence suggests that, in fact, anti-hypoxia drugs reducing Hif-1α levels can slow axial elongation (99), which points towards a possible relationship between myopia, hypoxia and Hif-1α. This relationship has also been described in genetic analyses revealing a moderate involvement of the Hif-1α signaling pathway in myopia (98, 100), However, tree shrews induced with myopia do not exhibit changes in scleral Hif-1α mRNA expression (101, 102), and guinea pigs with induced myopia show reduced scleral Hif-1α mRNA, highlighting the need for additional work in this field (102).

## 6. Vascular reactivity in myopia

The presence of structural and hemodynamic changes in the retinal vasculature in myopia suggests that myopic eyes might be suffering from an abnormal vascular function before degenerative changes occur (1, 54, 103–108). However, assessing ocular blood flow under normal conditions is not sufficient to detect vascular dysfunction. Retinal vascular function is assessed using provocation tests, indispensable to evaluate retinal reactivity and autoregulation (109–115). Autoregulation is the inherent local mechanism that ensures sufficient and stable blood flow under changing conditions to preserve adequate function of the surrounding tissues (116). Vascular regulation or vasoreactivity can be assessed by quantifying blood flow or vessel diameter changes that occur in response to flicker light (metabolic autoregulation), variations in the concentration of breathing oxygen (metabolic), or changes in systemic and intraocular pressure (shear-dependent and myogenic) (113, 117–120). Both the ocular and cerebral circulation exhibit an autoregulatory capacity (110, 117, 121–124). Three studies have evaluated the vascular reactivity profile of myopic eyes to date, and they used hypercapnia (increased pCO_2_) and hyperoxia (increased p0_2_) as provocation tests to assess the response from the retinal vasculature under stress conditions (125–127). Under room-air conditions prior to the provocation test, myopic and non-myopic eyes exhibited comparable systemic and ocular perfusion pressures –myopes exhibiting lower central retinal artery and choroidal blood flow. After inducing hypercapnia, myopic but not emmetropic eyes showed an increase in mean arterial pressure, along with a greater choroidal blood flow response, suggesting that myopes had a significantly lower resting choroidal flow that was highly responsive to CO_2_ (125, 126). These results insinuate an altered autoregulation ability in myopic eyes that, due to the increase mean arterial pressure observed, may lie in an autonomic dysregulation. Interestingly, there is evidence that eyes with pathological myopia eyes have comparable retinal vascular reactivity, suggesting that the retinal oxygen consumption, but not the choroidal, is altered in high-myopic eyes (128).

## 7. Molecular and cellular evidence of vascular changes in myopia

In order to understand the nature of the architectural and functional vascular described in myopia, it is important to comprehend the molecular and cellular changes taking place. The most extensive genetic myopia pathway analyses to date are clinical studies (meta-GWAS from 23andMe and the Consortium of Refractive Error And Myopia, CREAM) (129–134) and studies in common marmosets (*Callithrix jacchus*), a well-established non-human primate model of myopia (18).

Genetic meta-GWAS studies using human specimens have identified several signaling pathways involved in myopia, many of which were previously known, such as the extracellular matrix and ion channel pathways, while others were new, like those involved in angiogenesis. The following genes have been recently identified in meta-GWAS studies and found to have a role in vascular homeostasis: BMP2 and BMP4, CD34, CD55, Flt-1 and TGF-β receptor 1. BMP2 and BMP4 bone morphogenetic proteins (BMPs), named for their bone and cartilage formation ability (135, 136), are increasingly recognized as multifunctional regulators of angiogenesis. BMP2 has a pathological role in the development of vascular inflammation (137, 138), and induces retinal endothelial cell barrier dysfunction in diabetic macular edema and pathological retinal neovascularization (135). Over-expression of BMP4 inhibits experimental choroidal neovascularization by modulating VEGF and MMP-9 (139). CD34 is a transmembrane protein expressed in endothelial cells that promotes the formation of invasive vessels during neovascularization (140). The membrane-bound complement regulator CD55 is highly expressed in the retinal vascular endothelium (141), and significantly decreased in hyperoxic retinas (142). VEGF receptor–1 (also known as Flt-1) is needed for adequate blood vessel patterning on retinal astrocytes and can modulate VEGF-A activation of endothelial cells (143, 144). Flt-1 has unique and important roles in coordinating endothelial sprouting (145, 146), blood vessel anastomosis (147), and genetic loss of *flt-1* leading to vascular overgrowth and reduced network complexity (148). TGF-β receptor 1 inhibits and deep vascular plexus formation (149, 150), and its endothelial loss leads to aberrant contractile pericyte differentiation and hemorrhagic vascular malformations, and is essential for maintaining the integrity of mature vessels (151).

The analysis of retinal transcriptomes in marmosets induced with myopia has identified major molecular pathways activated during myopic eye growth (18). Some of the key pathways described are involved in vascular signaling and include the beta-adrenergic pathway, cyclic adenosine monophosphate (cAMP), Ca^2+^, relaxin (152), G protein-coupled receptor and nNOS. Beta-adrenergic signaling, for instance, is involved in hypoxia (153); there is a 90% increase in noradrenaline levels during hypoxia (154, 155), and beta-adrenorceptor activation is followed by an upregulation of hypoxia-inducible factor-1α (HIF-1α) and vascular endothelial growth factor (VEGF), both involved in neovascularization (156, 157). The cAMP pathway regulates neuronal, vascular, and inflammatory components of diabetic retinopathy (158). Calcium signaling is involved in capillary - but not arteriole - retinal blood flow as seen by the active dilation observed following astroglial Ca^2+^ signaling (159, 160). Relaxin, a peptide found at high concentrations during pregnancy, is found in endothelial and smooth muscle cells in arteries and veins, supporting its vasodilating role (161–163). G protein-coupled receptors 91 and 81 (GPR91and GPR81), localized in ganglion cells and Muller cells respectively, are involved in the pathogenesis of diabetic retinopathy (DR) and hypoxic retinal diseases such as retinopathy of prematurity (ROP), inner vascular network development and restoration of the vasculature in response to injury (152, 164). The neuronal isoform of nitric oxide (nNOS) is present in the vascular endothelium and contributes to the maintenance of homeostasis in the cardiovascular system. NO contributes to both retinal and choroidal neovascularization (165).

In terms of vascular cellular anatomy, the neurovascular interplay between neuronal, vascular, and glial cells, which is crucial for retinal structural and nutritional support and ion and neurotransmitter homeostasis, appears to be affected by myopia. Marmosets with induced myopia show a lower astrocyte density, increased GFAP-immunopositive staining, lower peripheral capillary branching, and increased numbers of string vessels compared to controls. These changes suggest an activation and reorganization of the astrocyte and vascular templates during myopia development and progression (166). Whether or not these adaptations are beneficial or harmful to the developing retina remains to be investigated.

## 8. Myopia-associated conditions showing vascular alterations

Myopic macular degeneration, glaucoma, choroidal neovascularization, retinal detachment, posterior staphyloma and cataract amongst the most prevalent myopia complications (167). In this section we review the vascular features of these myopia-associated conditions to help understand the vascular nature of myopia.

### 8.1. Myopic macular degeneration

Myopic maculopathy is the most common cause of vision loss in myopic eyes (5, 168), but its etiology remains unclear. The distinctive elongation and deformation of the myopic eye, along with its characteristic neovascularization, suggest that vascular pathways likely contribute to the degenerative process. Evidence for a vascular etiology of myopic maculopathy include a significant choroid thinning, enlarged foveal avascular zone, smaller choriocapillaris flow area, vascular dropout, lower fractal dimension, and a more profound decrease in deep but not superficial retinal capillary plexus density, suggesting that microvascular alterations appear crucial for myopic maculopathy (169–171). In addition, choroidal watershed zones, which are areas situated at the edge of end-arteries usually exhibiting a delayed choroidal filling, have been proposed to play a vascular role in the etiology of myopic maculopathy (172).

### 8.2. Glaucoma

Glaucoma is a complex neuropathy that preferentially affects the ganglion cell complex (GCC) (173) and exhibits features of vascular etiology and dysfunction (174). The relationship between myopia and glaucoma has been confirmed by several large population-based studies (175–180). Yet, the nature of the myopia-glaucoma relationship remains unknown. Low ocular perfusion has been identified as a risk factor for glaucoma progression independent of intraocular pressure (181–183). Both glaucomatous and myopic eyes show parallel vascular changes: retinal microvasculature attenuation (184), decreased capillary density (63, 66, 184–186), and reduced retinal and ONH blood flow and vascular dysregulation (56, 57, 65, 68, 89, 91, 187–189). In fact, the longer the axial eye length and the thinner the ocular wall in glaucoma patients, the greater the retinal microcirculation reduction (190). In addition, when the vascular features of glaucomatous patients with and without myopia were compared, myopic glaucomatous eyes exhibited greater vascular changes than non-myopic glaucomatous eyes: larger reductions in choroidal blood flow and velocity (191), lower macular and peripapillary capillary density (184, 192), and impaired peripapillary vasoreactivity (127). Therefore, it has been hypothesized that the relationship between myopia and glaucoma might partly be vascular in nature, specifically microvascular, and may be present before the glaucomatous degeneration is evident. In addition, the study of vascular reactivity in glaucomatous patients with and without high myopia has confirmed that the retinal vasoreactivity of the peripapillary capillaries is compromised in glaucomatous eyes with high myopia (127).

### 8.3. Choroidal neovascularization

Choroidal neovascularization (CNV) is characterized by an atypical choroidal vasculature growth into the retinal pigment epithelium potentially leading to fluid and blood accumulation in the macula (50). Eyes with lower foveolar choroidal blood volume and flow have been identified to be at a higher risk of developing CNV. This reduced choroidal blood supply appears greater than any changes observed in eyes without CNV, suggesting that alterations in the foveal choroidal circulation might precede be part of CNV etiology (193). In addition, the choroidal thinning and capillary density reduction observed in degenerative pathological myopia is believed to trigger RPE and glial cells hypoxia, resulting in an upregulation of VEGF expression (194).

### 8.4. Retinal detachment

The most prevalent form of neurosensory retina separation from the retinal pigment epithelium (RPE) is the rhegmatogenous retinal detachment. This type of detachment disrupts the retinal vasculature leading to smaller vascular diameters, tortuosity, and vascular density (195). Capillary dilatation, hyperpermeability and fluorescein leakage have also been observed with fluorescein angiography on the detached retina, and have been proposed to originate from tissue hypoxia (196, 197).

### 8.5. Posterior staphyloma

Peripapillary posterior staphyloma (PPS) is one of six types of posterior staphylomas identified in degenerative myopia (198). Eyes with PPS have an increased macular vessel density in the deep plexus, reduced macular choriocapillaris and radial peripapillary capillary density, and thinner choroids (199). In addition, a retrospective study also identified reduced choriocapillaris flow and thinner subfoveal choroidal thickness, confirming that eyes with posterior staphyloma have thinner choroids and lower perfusion (200).

### 8.6. Cataract

A relationship between lens opacity and hypertension was identified in the initial cross-sectional phase of the Beaver Dam Eye Study (201). However, this relationship was not confirmed once the longitudinal 5-year Beaver Dam Eye Study was completed (202). There is, however, evidence of lower ocular blood velocity in cataract patients that requires further evaluation to understand the nature of the changes (203).

## 9. Conclusion

Adequate blood flow is fundamental for tissue homeostasis (204). In view of the findings described in the literature and discussed in this review, studying the haemodynamics and vascular autoregulation features of healthy myopic eyes may be crucial to identify early markers of associated degeneration and help develop novel vascular interventions to preserve the health of myopic eyes.

## Author contributions

AB-P conceived, designed, wrote and reviewed the manuscript.

## Conflict of interest

The author declares that the research was conducted in the absence of any commercial or financial relationships that could be construed as a potential conflict of interest.

## Publisher’s note

All claims expressed in this article are solely those of the authors and do not necessarily represent those of their affiliated organizations, or those of the publisher, the editors and the reviewers. Any product that may be evaluated in this article, or claim that may be made by its manufacturer, is not guaranteed or endorsed by the publisher.

## References

[1] Ohno-Matsui K Akiba M Ishibashi T Moriyama M. Observations of vascular structures within and posterior to sclera in eyes with pathologic myopia by swept-source optical coherence tomography. Invest Ophthalmol Vis Sci. (2012) 53:7290–8. doi: 10.1167/iovs.12-10371, PMID: 23033385

[2] Vongphanit J Mitchell P Wang JJ. Prevalence and progression of myopic retinopathy in an older population. Ophthalmology. (2002) 109:704–11. doi: 10.1016/S0161-6420(01)01024-7, PMID: 11927427

[3] Saw SM. How blinding is pathological myopia? Br J Ophthalmol. (2006) 90:525–6. doi: 10.1136/bjo.2005.087999, PMID: 16622078 PMC1857043

[4] Ruiz-Moreno JM Arias L Montero JA Carneiro A Silva R. Intravitreal anti-Vegf therapy for choroidal neovascularisation secondary to pathological myopia: 4-year outcome. Br J Ophthalmol. (2013) 97:1447–50. doi: 10.1136/bjophthalmol-2012-302973, PMID: 24026146

[5] Neelam K Cheung CM Ohno-Matsui K Lai TY Wong TY. Choroidal neovascularization in pathological myopia. Prog Retin Eye Res. (2012) 31:495–525. doi: 10.1016/j.preteyeres.2012.04.001, PMID: 22569156

[6] Mutti DO Semina E Marazita M Cooper M Murray JC Zadnik K. Genetic loci for pathological myopia are not associated with juvenile myopia. Am J Med Genet. (2002) 112:355–60. doi: 10.1002/ajmg.10683, PMID: 12376937

[7] Morgan IG Ohno-Matsui K Saw SM. Myopia. Lancet. (2012) 379:1739–48. doi: 10.1016/s0140-6736(12)60272-4 22559900

[8] Ohno-Matsui K Kawasaki R Jonas JB Cheung CMG Saw S-M Verhoeven VJM. International photographic classification and grading system for myopic maculopathy. Am J Ophthalmol. (2015) 159:877–83.e7. doi: 10.1016/j.ajo.2015.01.022, PMID: 25634530

[9] Curtin BJ. The Myopias: Basic Science and Clinical Management. Philadelphia: Harper and Row (1985).

[10] Vutipongsatorn K Yokoi T Ohno-Matsui K. Current and emerging pharmaceutical interventions for myopia. Br J Ophthalmol. (2019) 103:1539. doi: 10.1136/bjophthalmol-2018-313798, PMID: 31097440

[11] Holden BA Fricke TR Wilson DA Jong M Naidoo KS Sankaridurg P. Global prevalence of myopia and high myopia and temporal trends from 2000 through 2050. Ophthalmology. (2016) 123:1036–42. doi: 10.1016/j.ophtha.2016.01.006, PMID: 26875007

[12] Smith EL III. Prentice award lecture 2010: a case for peripheral optical treatment strategies for myopia. Optom Vis Sci. (2011) 88:1029–44. doi: 10.1097/OPX.0b013e3182279cfa, PMID: 21747306 PMC3371664

[13] Faria-Ribeiro M Queirós A Lopes-Ferreira D Jorge J González-Méijome JM. Peripheral refraction and retinal contour in stable and progressive myopia. Optom Vis Sci. (2013) 90:9–15. doi: 10.1097/OPX.0b013e318278153c, PMID: 23208195

[14] Ashby RS Schaeffel F. The effect of ambient illuminance on the development of deprivation myopia in chicks. Invest Ophthalmol Vis Sci. (2009) 50:5348–54. doi: 10.1167/iovs.09-3419, PMID: 19516016

[15] Smith EL III Hung LF Huang J. Effects of high ambient lighting on the development of form-deprivation myopia in infant Rhesus monkeys. Invest Ophthalmol Vis Sci. (2012) 53:421–8. doi: 10.1167/iovs.11-8652 22169102 PMC3292375

[16] Rose KA Morgan IG Ip J Kifley A Huynh S Smith W. Outdoor activity reduces the prevalence of myopia in children. Ophthalmol. (2008) 115:1279–85. doi: 10.1016/j.ophtha.2007.12.019, PMID: 18294691

[17] Dirani M Tong L Gazzard G Zhang X Chia A Young TL. Outdoor activity and myopia in Singapore teenage children. Br J Ophthalmol. (2009) 93:997–1000. doi: 10.1136/bjo.2008.150979, PMID: 19211608

[18] Tkatchenko TV Troilo D Benavente-Perez A Tkatchenko AV. Gene expression in response to optical defocus of opposite signs reveals bidirectional mechanism of visually guided eye growth. PLoS Biol. (2018) 16:e2006021. doi: 10.1371/journal.pbio.2006021 30300342 PMC6177118

[19] Chakraborty R Park HN Hanif AM Sidhu CS Iuvone PM Pardue MT. On pathway mutations increase susceptibility to form-deprivation myopia. Exp Eye Res. (2015) 137:79–83. doi: 10.1016/j.exer.2015.06.009, PMID: 26072023 PMC4523446

[20] Forrester JV Dick AD McMenamin PG Roberts F Pearlman E. The Eye: Basic Sciences in Practice. Edinburgh, United Kingdom: Elsevier (2001).

[21] Yu DY Cringle SJ. Oxygen distribution and consumption within the retina in vascularised and avascular retinas and in animal models of retinal disease. Prog Retin Eye Res. (2001) 20:175–208. doi: 10.1016/S1350-9462(00)00027-6, PMID: 11173251

[22] Hayreh SS In: Dartt D, editor. Encyclopedia of the Eye. United States: Academic Press (2010). 43:1–8.

[23] Delaey C Van de Voorde J. Regulatory mechanisms in the retinal and choroidal circulation. Ophthalmic Res. (2000) 32:249–56. doi: 10.1159/000055622, PMID: 11015035

[24] Flammer J Orgüla S Costab V Orzalesic N Krieglsteind G Metzner Serrae K. The impact of ocular blood flow in glaucoma. Prog Retin Eye Res. (2002) 21:359–93. doi: 10.1016/S1350-9462(02)00008-3, PMID: 12150988

[25] Bron A Tripathi R Tripathi B. Wolff's Anatomy of the Eye and Orbit. 8th ed. London: Chapman & Hall Medical (1997).

[26] Hayreh SS. Blood flow in the optic nerve head and factors that may influence it. Prog Retin Eye Res. (2001) 20:595–624. doi: 10.1016/S1350-9462(01)00005-2, PMID: 11470452

[27] Akyol N Kukner A Ozdemir T Esmerligil S. Choroidal and retinal blood flow changes in degenerative myopia. Can J Ophthalmol. (1996) 31:113–9. PMID: 8743218

[28] Dimitrova G Tamaki Y Kato S Nagahara M. Retrobulbar circulation in myopic patients with or without myopic choroidal neovascularisation. Br J Ophthalmol. (2002) 86:771–3. doi: 10.1136/bjo.86.7.771, PMID: 12084747 PMC1771207

[29] Wu H Seet B Yap E Saw S Lim T Chia K. Does education explain ethnic differences in myopia prevalence? A population-based study of Young adult males in Singapore. Optom Vis Sci. (2001) 78:234–9. doi: 10.1097/00006324-200104000-00012, PMID: 11349931

[30] Fitzgerald M Wildsoe C Anton R. Temporal relationship of choroidal blood flow and thickness changes during recovery from form deprivation myopia in chicks. Exp Eye Res. (2001) 74:561–70. doi: 10.1006/exer.2002.1142 12076077

[31] Fitzgerald MEC Tolley E Jackson B Zagvazdin YS Cuthbertson SL Hodos W. Anatomical and functional evidence for progressive age-related decline in parasympathetic control of choroidal blood flow in pigeons. Exp Eye Res. (2005) 81:478–91. doi: 10.1016/j.exer.2005.03.008, PMID: 15935343

[32] Wildsoet C Wallman J. Choroidal and scleral mechanisms of compensation for spectacle lenses in chicks. Vis Res. (1995) 35:1175–94. doi: 10.1016/0042-6989(94)00233-C, PMID: 7610579

[33] Jin N Stjernschantz J. Regional blood flow in the myopic Chick eye during and after form deprivation: a study with radioactively-labelled microspheres. Exp Eye Res. (2000) 71:233–8. doi: 10.1006/exer.2000.0871, PMID: 10973732

[34] Riva C Ross B Benedek GB. Laser Doppler measurements of blood flow in capillary tubes and retinal arteries. Investig Ophthalmol. (1972) 11:936–44. PMID: 4634958

[35] Olafsdottir OB Eliasdottir TS Kristjansdottir JV Hardarson SH Stefánsson E. Retinal vessel oxygen saturation during 100% oxygen breathing in healthy individuals. PLoS One. (2015) 10:e0128780. doi: 10.1371/journal.pone.0128780, PMID: 26042732 PMC4456093

[36] de Carlo TE Romano A Waheed NK Duker JS. A review of optical coherence tomography angiography (Octa). Int J Retina Vitreous. (2015) 1:5. doi: 10.1186/s40942-015-0005-8 27847598 PMC5066513

[37] Mo S Krawitz B Efstathiadis E Geyman L Weitz R Chui TY. Imaging foveal microvasculature: optical coherence tomography angiography versus adaptive optics scanning light ophthalmoscope fluorescein angiography. Invest Ophthalmol Vis Sci. (2016) 57:130–40. doi: 10.1167/iovs.15-18932 PMC4968918 27409463

[38] Senarathna J Rege A Li N Thakor NV. Laser speckle contrast imaging: theory, instrumentation and applications. IEEE Rev Biomed Eng. (2013) 6:99–110. doi: 10.1109/RBME.2013.2243140, PMID: 23372086

[39] Zion IB Harris A Siesky B Shulman S McCranor L Garzozi HJ. Pulsatile ocular blood flow: relationship with flow velocities in vessels supplying the retina and choroid. Br J Ophthalmol. (2007) 91:882–4. doi: 10.1136/bjo.2006.108340, PMID: 17576711 PMC1955661

[40] Garhofer G Werkmeister R Dragostinoff N Schmetterer L. Retinal blood flow in healthy Young subjects. Invest Ophthalmol Vis Sci. (2012) 53:698–703. doi: 10.1167/iovs.11-8624, PMID: 22247463

[41] Riva CE Grunwald JE Sinclair SH Petrig BL. Blood velocity and volumetric flow rate in human retinal vessels. Invest Ophthalmol Vis Sci. (1985) 26:1124–32. PMID: 4019103

[42] Leitgeb RA Werkmeister RM Blatter C Schmetterer L. Doppler optical coherence tomography. Prog Retin Eye Res. (2014) 41:26–43. doi: 10.1016/j.preteyeres.2014.03.004, PMID: 24704352 PMC4073226

[43] Emeterio Nateras OS Harrison JM Muir ER Zhang Y Peng Q Chalfin S. Choroidal blood flow decreases with age: an Mri study. Curr Eye Res. (2014) 39:1059–67. doi: 10.3109/02713683.2014.892997, PMID: 24655028 PMC4241237

[44] Burgansky-Eliash Z Lowenstein A Neuderfer M Kesler A Barash H Nelson DA. The correlation between retinal blood flow velocity measured by the retinal function imager and various physiological parameters. Ophthalmic Surg Lasers Imaging Retina. (2013) 44:51–8. doi: 10.3928/23258160-20121221-13, PMID: 23418734

[45] Yu J Jiang C Wang X Zhu L Gu R Xu H. Macular perfusion in healthy Chinese: an optical coherence tomography angiogram study. Invest Ophthalmol Vis Sci. (2015) 56:3212–7. doi: 10.1167/iovs.14-16270, PMID: 26024105 PMC4455309

[46] Yu J Gu R Zong Y Xu H Wang X Sun X. Relationship between retinal perfusion and retinal thickness in healthy subjects: an optical coherence tomography angiography study. Invest Ophthalmol Vis Sci. (2016) 57:204–10. doi: 10.1167/iovs.15-18630, PMID: 27409474 PMC4968770

[47] Lin Y Jiang H Liu Y Rosa Gameiro G Gregori G Dong C. Age-related alterations in retinal tissue perfusion and volumetric vessel density. Invest Ophthalmol Vis Sci. (2019) 60:685–93. doi: 10.1167/iovs.18-25864, PMID: 30786280 PMC6383727

[48] Wei Y Jiang H Shi Y Qu D Gregori G Zheng F. Age-related alterations in the retinal microvasculature, microcirculation, and microstructure. Invest Ophthalmol Vis Sci. (2017) 58:3804–17. doi: 10.1167/iovs.17-21460, PMID: 28744554 PMC5527847

[49] Fitzgerald MEC Tolley E Frase S Zagvazdin Y Miller RF Hodos W. Functional and morphological assessment of age-related changes in the choroid and outer retina in pigeons. Vis Neurosci. (2001) 18:299–317. doi: 10.1017/S0952523801182143, PMID: 11417804

[50] Chirco KR Sohn EH Stone EM Tucker BA Mullins RF. Structural and molecular changes in the aging choroid: implications for age-related macular degeneration. Eye (Lond). (2017) 31:10–25. doi: 10.1038/eye.2016.216, PMID: 27716746 PMC5233940

[51] Kur J Newman EA Chan-Ling T. Cellular and physiological mechanisms underlying blood flow regulation in the retina and choroid in health and disease. Prog Retin Eye Res. (2012) 31:377–406. doi: 10.1016/j.preteyeres.2012.04.004 22580107 PMC3418965

[52] Avetisov ES Savitskaya NF. Some features of ocular microcirculation in myopia. Ann Ophthalmol. (1977) 9:1261–4. PMID: 921147

[53] Bal'zhanova AB. Fluorescence angiography in assessing choroidal circulation in highly progressive myopia. Vestn oftalmol. (1978) 1:25–30. 636148

[54] Lim LS Cheung CY Lin X Mitchell P Wong TY Mei-Saw S. Influence of refractive error and axial length on retinal vessel geometric characteristic. Invest Ophthalmol Vis Sci. (2011) 52:669–78s. doi: 10.1167/iovs.10-6184, PMID: 20847122

[55] Sun C Chen T Cong J Wu X Wang J Yuan Y. Changes in retinal vascular bifurcation in eyes with myopia. BMC Ophthalmol. (2022) 22:408. doi: 10.1186/s12886-022-02629-y 36271390 PMC9585760

[56] Benavente-Perez A Hosking SL Logan NS Broadway DC. Ocular blood flow measurements in healthy human myopic eyes. Graefes Arch Clin Exp Ophthalmol. (2010) 248:1587–94. doi: 10.1007/s00417-010-1407-9 20502909

[57] Shimada N Ohno-Matsui K Harino S Yoshida T Yasuzumi K Kojima A. Reduction of retinal blood flow in high myopia. Graefes Arch Clin Exp Ophthalmol. (2004) 242:284–8. doi: 10.1007/s00417-003-0836-0 14722781

[58] Karczewicz D Modrzejewska M. Blood flow in eye arteries assessed by Doppler ultrasound in patients with myopia. Klin Ocz. (2004) 106:211–3. 15510503

[59] Mamikonian VR Shmeleva-Demir OA Kharlap SI Andzhelova DV Kazarian EE Makashova NV. Hemodynamic changes in myopia of different degrees. Vestn oftalmol. (2013) 129:24–7. PMID: 24624798

[60] Mo J Duan A Chan S Wang X Wei W. Vascular flow density in pathological myopia: an optical coherence tomography angiography study. BMJ Open. (2017) 7:e013571. doi: 10.1136/bmjopen-2016-013571, PMID: 28159853 PMC5294002

[61] Yang Y Wang J Jiang H Yang X Feng L Hu L. Retinal microvasculature alteration in high myopia. Invest Ophthalmol Vis Sci. (2016) 57:6020–30. doi: 10.1167/iovs.16-19542, PMID: 27820633

[62] Li Y Miara H Ouyang P Jiang B. The comparison of regional Rnfl and fundus vasculature by Octa in Chinese myopia population. J Ophthalmol. (2018) 2018:10. doi: 10.1155/2018/3490962 PMC5830980 29651341

[63] Su L Ji YS Tong N Sarraf D He X Sun X. Quantitative assessment of the retinal microvasculature and Choriocapillaris in myopic patients using swept-source optical coherence tomography angiography. Graefes Arch Clin Exp Ophthalmol. (2020) 258:1173–80. doi: 10.1007/s00417-020-04639-2, PMID: 32144487

[64] Min CH Al-Qattan HM Lee JY Kim J-G Yoon YH Kim YJ. Macular microvasculature in high myopia without pathologic changes: an optical coherence tomography angiography study. Korean J Ophthalmol. (2020) 34:106–12. doi: 10.3341/kjo.2019.0113, PMID: 32233143 PMC7105786

[65] Wang X Kong X Jiang C Li M Yu J Sun X. Is the Peripapillary retinal perfusion related to myopia in healthy eyes? A prospective comparative study. BMJ Open. (2016) 6:e010791. doi: 10.1136/bmjopen-2015-010791, PMID: 26969645 PMC4800142

[66] Al-Sheikh M Phasukkijwatana N Dolz-Marco R Rahimi M Iafe NA Freund KB. Quantitative Oct angiography of the retinal microvasculature and the Choriocapillaris in myopic eyes. Invest Ophthalmol Vis Sci. (2017) 58:2063–9. doi: 10.1167/iovs.16-21289, PMID: 28388703

[67] Wang T Li H Zhang R Yu Y Xiao X Wu C. Evaluation of retinal vascular density and related factors in youth myopia without maculopathy using Octa. Sci Rep. (2021) 11:15361. doi: 10.1038/s41598-021-94909-8 34321564 PMC8319333

[68] Qu D Lin Y Jiang H Shao Y Shi Y Airen S. Retinal nerve Fiber layer (Rnfl) integrity and its relations to retinal microvasculature and microcirculation in myopic eyes. Eye Vis. (2018) 5:25. doi: 10.1186/s40662-018-0120-3 PMC6190551 30349842

[69] Lim LS Cheung CY Lin X Mitchell P Wong TY Mei-Saw S. Influence of refractive error and axial length on retinal vessel geometric characteristics. Invest Ophthalmol Vis Sci. (2011) 52:669–78. doi: 10.1167/iovs.10-6184, PMID: 20847122

[70] Man RE Sasongko MB Xie J Best WJ Noonan JE Lo TC. Decreased retinal capillary flow is not a mediator of the protective myopia-diabetic retinopathy relationship. Invest Ophthalmol Vis Sci. (2014) 55:6901–7. doi: 10.1167/iovs.14-15137, PMID: 25270188

[71] Wallman J Wildsoet C Xu A Gottlieb MD Nickla D Marran L. Moving the retina: choroidal modulation of refractive state. VR. (1995) 35:37–70. 10.1016/0042-6989(94)e0049-q 7839608

[72] Hirata A Negi A. Morphological changes of Choriocapillaris in experimentally induced Chick Myopa. Graefes Arch Clin Exp Ophthalmol. (1998) 236:132–7. 9498124 10.1007/s004170050053

[73] Fitzgerald MEC Wildsoet CF Reiner A. Choroidal blood flow and thickness changes during recovery from form deprivation myopia in chicks – are they causally related? ARVO. (1998) 39:S868. 10.1006/exer.2002.1142 12076077

[74] Troilo D Nickla DL Wildsoet CW. Choroidal thickness changes during altered eye growth and refractive state in a primate. Investig Ophthalmol Vis Sci. (2000) 41:1249–58. PMID: 10798638

[75] Shih YF Fitzgerald MEC Norton TT Gamlin PDR Hodos W Reiner A. Reduction in choroidal blood flow occurs in chicks wearing goggles that induce eye growth toward myopia. Curr Eye Res. (1993) 12:219–27. 8482110 10.3109/02713689308999467 PMC4460565

[76] Hirata A Negi A. Lacquer crack lesions in experimental Chick myopia. Graefes Arch Clin Exp Ophthalmol. (1998) 236:138–45. 9498125 10.1007/s004170050054

[77] Fitzgerald MEC Wildsoet CF Reiner A. Temporal relationship of choroidal blood flow and thickness changes during recovery from form deprivation myopia in chicks. Exp Eye Res. (2002) 74:561–70. doi: 10.1006/exer.2002.1142, PMID: 12076077

[78] Harb E Hyman L Gwiazda J Marsh-Tootle W Zhang Q Hou W. Choroidal thickness profiles in myopic eyes of Young adults in the correction of myopia evaluation trial cohort. Am J Ophthalmol. (2015) 160:62–71.e2. doi: 10.1016/j.ajo.2015.04.018, PMID: 25896460 PMC4465039

[79] Hung LF Wallman J Smith EL. Vision-dependent changes in the choroidal thickness of macaque monkeys. Invest Ophthalmol Vis Sci. (2000) 41:1259–69. 10798639

[80] Howlett MH McFadden SA. Spectacle Lens compensation in the pigmented Guinea pig. Vis Res. (2009) 49:219–27. doi: 10.1016/j.visres.2008.10.008, PMID: 18992765

[81] Read SA Alonso-Caneiro D Vincent SJ Collins MJ. Longitudinal changes in choroidal thickness and eye growth in childhood. Invest Ophthalmol Vis Sci. (2015) 56:3103–12. doi: 10.1167/iovs.15-16446, PMID: 26024094

[82] Read SA Alonso-Caneiro D Vincent SJ Collins MJ. Peripapillary choroidal thickness in childhood. Exp Eye Res. (2015) 135:164–73. doi: 10.1016/j.exer.2015.03.002, PMID: 25749004

[83] Liu L Zhu C Yuan Y Hu X Chen C Zhu H. Three-dimensional choroidal vascularity index in high myopia using swept-source optical coherence tomography. Curr Eye Res. (2022) 47:484–92. doi: 10.1080/02713683.2021.2006236, PMID: 35130815

[84] Alshareef RA Khuthaila MK Goud A Vupparaboina KK Jana S Chhablani J. Subfoveal choroidal vascularity in myopia: evidence from spectral-domain optical coherence tomography. Ophthalmic Surg Lasers Imaging Retina. (2017) 48:202–7. doi: 10.3928/23258160-20170301-02, PMID: 28297031

[85] Hitchings R. The ocular pulse. Br J Ophthalmol. (1991) 75:65. doi: 10.1136/bjo.75.2.65, PMID: 1995044 PMC504114

[86] Berisha F Findl O Lasta M Kiss B Schmetterer L. A study comparing ocular pressure pulse and ocular fundus pulse in dependence of axial eye length and ocular volume. Acta Ophthalmol. (2010) 88:766–72. doi: 10.1111/j.1755-3768.2009.01577.x, PMID: 20337602

[87] James CB Trew DR Clark K Smith SE. Factors influencing the ocular pulse – axial length. Graefes Arch Clin Exp Ophthalmol. (1991) 229:341–4. doi: 10.1007/bf00170692 1916321

[88] Zhang S Zhang G Zhou X Xu R Wang S Guan Z. Changes in choroidal thickness and choroidal blood perfusion in Guinea pig myopia. Invest Ophthalmol Vis Sci. (2019) 60:3074–83. doi: 10.1167/iovs.18-26397, PMID: 31319419

[89] Scherm P Pettenkofer M Maier M Lohmann CP Feucht N. Choriocapillary blood flow in myopic patients measured with Oct angiography. Invest Ophthalmol Vis Sci. (2018) 59:2821. 10.3928/23258160-20190503-13 31100167

[90] Ning J Joshi N Franchi-Pereira R Benavente-Perez A. Longitudinal evaluation of choroidal thickness and ocular perfusion pressure in progressing Myopes, baseline data. Invest Ophthalmol Vis Sci. (2017) 58:1106.

[91] Cheng W Song Y Gao X Lin F Li F Wang P. Axial length and Choriocapillaris flow deficits in non-pathological high myopia. Am J Ophthalmol. (2022) 244:68–78. doi: 10.1016/j.ajo.2022.08.005, PMID: 35970207

[92] Wang Y Chen S Lin J Chen W Huang H Fan X. Vascular changes of the choroid and their correlations with visual acuity in pathological myopia. Invest Ophthalmol Vis Sci. (2022) 63:20. doi: 10.1167/iovs.63.12.20, PMID: 36378132 PMC9672896

[93] Zhang Z Qi Y Wei W Jin Z-B Wang W Duan A. Investigation of macular choroidal thickness and blood flow change by optical coherence tomography angiography after posterior scleral reinforcement. Front Med. (2021):8. doi: 10.3389/fmed.2021.658259 PMC8130341 34017847

[94] Lim LS Lim XH Tan L. Retinal vascular oxygen saturation and its variation with refractive error and axial length. Transl Vis Sci Technol. (2019) 8:22. doi: 10.1167/tvst.8.4.22, PMID: 31403000 PMC6685695

[95] Liu X He X Yin Y Zhang B Sun S Zhu J. Retinal oxygen saturation in 1461 healthy children aged 7-19 and its associated factors. Acta Ophthalmol. (2019) 97:287–95. doi: 10.1111/aos.14043, PMID: 30714353 PMC6590240

[96] Zheng Q Zong Y Li L Huang X Lin L Yang W. Retinal vessel oxygen saturation and vessel diameter in high myopia. Ophthalmic Physiol Opt. (2015) 35:562–9. doi: 10.1111/opo.12223, PMID: 26303449

[97] Liu Y Wang L Xu Y Pang Z Mu G. The influence of the choroid on the onset and development of myopia: from perspectives of choroidal thickness and blood flow. Acta Ophthalmol. (2021) 99:730–8. doi: 10.1111/aos.14773, PMID: 33550704

[98] Brown DM Mazade R Clarkson-Townsend D Hogan K Datta Roy PM Pardue MT. Candidate pathways for retina to scleral signaling in refractive eye growth. Exp Eye Res. (2022) 219:109071. doi: 10.1016/j.exer.2022.109071, PMID: 35447101 PMC9701099

[99] Wu H Chen W Zhao F Zhou Q Reinach PS Deng L. Scleral hypoxia is a target for myopia control. Proc Natl Acad Sci. (2018) 115:E7091–100. doi: 10.1073/pnas.1721443115, PMID: 29987045 PMC6064999

[100] Zhao F Zhang D Zhou Q Zhao F He M Yang Z. Scleral Hif-1α is a prominent regulatory candidate for genetic and environmental interactions in human myopia pathogenesis. EBioMedicine. (2020) 57:102878. doi: 10.1016/j.ebiom.2020.102878, PMID: 32652319 PMC7348000

[101] Guo L Frost MR Siegwart JT Jr Norton TT. Gene expression signatures in tree shrew sclera during recovery from minus-Lens Wear and during plus-Lens Wear. Mol Vis. (2019) 25:311–28. PMID: 31341380 PMC6610222

[102] Guo L Frost MR He L Siegwart JT Jr Norton TT. Gene expression signatures in tree shrew sclera in response to three Myopiagenic conditions. Invest Ophthalmol Vis Sci. (2013) 54:6806–19. doi: 10.1167/iovs.13-12551, PMID: 24045991 PMC3805087

[103] Benavente-Perez A Hosking SL Logan NS Bansal D. Reproducibility-repeatability of choroidal thickness calculation using Oct. Optom Vis Sci. (2010) 87:867–72. doi: 10.1097/OPX.0b013e3181f3eced, PMID: 20818280

[104] Goldenberg D Moisseiev E Goldstein M Loewenstein A Barak A. Enhanced depth imaging optical coherence tomography: choroidal thickness and correlations with age, refractive error, and axial length. Ophthalmic Surg Lasers Imaging. (2012) 43:296–301. doi: 10.3928/15428877-20120426-02, PMID: 22589335

[105] Li XQ Larsen M Munch IC. Subfoveal choroidal thickness in relation to sex and axial length in 93 Danish university students. Invest Ophthalmol Vis Sci. (2011) 52:8438–41. doi: 10.1167/iovs.11-8108, PMID: 21917938

[106] Agawa T Miura M Ikuno Y Makita S Fabritius T Iwasaki T. Choroidal thickness measurement in healthy Japanese subjects by three-dimensional high-penetration optical coherence tomography. Graefes Arch Clin Exp Ophthalmol. (2011) 249:1485–92. 21556938 10.1007/s00417-011-1708-7

[107] Ohno-Matsui K Akiba M Modegi T Tomita M Ishibashi T Tokoro T. Association between shape of sclera and myopic Retinochoroidal lesions in patients with pathologic myopia. Invest Ophthalmol Vis Sci. (2012) 53:6046–61. doi: 10.1167/iovs.12-10161, PMID: 22879412

[108] Ohno-Matsui K Akiba M Moriyama M Ishibashi T Tokoro T Spaide RF. Imaging Retrobulbar subarachnoid space around optic nerve by swept-source optical coherence tomography in eyes with pathologic myopia. Invest Ophthalmol Vis Sci. (2011) 52:9644–50. doi: 10.1167/iovs.11-8597, PMID: 22076987

[109] Riva C. Rhythmic changes in velocity, volume and blood flow in the optic nerve head tissue. Microvasc Res. (1990) 40:36–45. doi: 10.1016/0026-2862(90)90005-C, PMID: 2144606

[110] Hafez A Bizzarro R Rivard M Trabut I Lovasik J Kergoat H. Reproducibility of retinal and optic nerve head perfusion measurements using scanning laser Doppler Flowmetry. Ophthalmic Surg Lasers Imaging. (2003) 34:422–32. doi: 10.3928/1542-8877-20030901-18, PMID: 14509472

[111] Schemetterer L Lexer F Findl LO Graselli U Eichler H Woltz M. The effect of inhalation of different mixtures of O2and Co2on ocular fundus pulsations. Exp Eye Res. (1996) 63:351–5. doi: 10.1006/exer.1996.0125 8944542

[112] Schmetterer L. Noninvasive investigations of the Normal ocular circulation in humans. Invest Ophthalmol Vis Sci. (1998) 39:1210. PMID: 9620081

[113] Hosking SL Harris A Chung HS Jonescu-Cuypers CP Kagemann L Roff Hilton EJ. Ocular Haemodynamic responses to induced hypercapnia and Hyperoxia in Glaucoma. Br J Ophthalmol. (2004) 88:406–11. doi: 10.1136/bjo.2002.008995, PMID: 14977778 PMC1772045

[114] Embleton S Hosking S Roff Hilton E Cunliffe I. Effect of senescence on ocular blood flow in the retina, neuroretinal rim and lamina cribrosa, using scanning laser Doppler flowmetry. Eye (Lond). (2002) 16:156–62. doi: 10.1038/sj.eye.6700100 11988816

[115] Logan N Gilmartin B Cox W. Ocular Volume and Blood Flow in Human Anisomyopia. Invest Ophthalmol Vis Sci. (2002) 43:199.

[116] Guyton AC. Evidence for tissue oxygen demand as the major factor causing autoregulation. Circ Res. (1964) 15:60–9. 14206321

[117] Riva CE Grunwald JE Petrig BL. Autoregulation of human retinal blood flow. An investigation with laser Doppler velocimetry. Invest Ophthalmol Vis Sci. (1986) 27:1706–12. PMID: 2947873

[118] Michelson G Groh M Gründler A. Regulation of ocular blood flow during increases of arterial blood pressure. Br J Ophthalmol. (1994) 78:461–5. doi: 10.1136/bjo.78.6.461, PMID: 8060930 PMC504824

[119] Kergoat H Faucher C. Effects of oxygen and Carbogen breathing on choroidal hemodynamics in humans. Invest Ophthalmol Vis Sci. (1999) 40:2906–11. PMID: 10549651

[120] Schumann J Orgül S Gugleta K Dubler B Flammer J. Interocular difference in progression of Glaucoma correlates with Interocular differences in Retrobulbar circulation. Am J Ophthalmol. (2000) 129:728–33. doi: 10.1016/S0002-9394(99)00481-X, PMID: 10926980

[121] Aaslid R Lindegaard KF Sorteberg W Nornes H. Cerebral autoregulation dynamics in humans. Stroke. (1989) 20:45–52. doi: 10.1161/01.STR.20.1.45, PMID: 2492126

[122] Riva C Hero M Titze P Petrig B. Autoregulation of human optic nerve head blood flow in response to acute changes in ocular perfusion pressure. Graefes Arch Clin Exp Ophthalmol. (1997) 235:618–26. doi: 10.1007/BF00946937, PMID: 9349945

[123] Longo A Geiser M Riva C. Posture changes and subfoveal choroidal blood flow. Investig Ophthalmol Vis Sci. (2004) 45:546–51. doi: 10.1167/iovs.03-0757, PMID: 14744897

[124] Riva CE Logean E Falsini B. Visually evoked Hemodynamical response and assessment of neurovascular coupling in the optic nerve and retina. Prog Retin Eye Res. (2005) 24:183–215. doi: 10.1016/j.preteyeres.2004.07.002, PMID: 15610973

[125] Benavente-Perez A Hosking SL Logan NS. Defective haemodynamic autoregulation associated with autonomic dysregulation in human myopia. Invest Ophthalmol Vis Sci. (2007) 48:1041.

[126] Benavente-Perez A Hosking SL Logan NS. Myopes exhibit reduced choroidal blood velocity which is highly responsive to hypercapnia. Invest Ophthalmol Vis Sci. (2008) 49:3581.

[127] Fan X Xu H Zhai R Sheng Q Sun Y Shao T. Peripapillary vascular reactivity in primary open-angle Glaucoma with high myopia by using optical coherence tomography angiography. Front Med. (2022) 9:850483. doi: 10.3389/fmed.2022.850483, PMID: 35372433 PMC8971362

[128] La Spina C Corvi F Bandello F Querques G. Static characteristics and dynamic functionality of retinal vessels in longer eyes with or without pathologic myopia. Graefes Arch Clin Exp Ophthalmol. (2016) 254:827–34. doi: 10.1007/s00417-015-3122-z 26245340

[129] Tedja MS Wojciechowski R Hysi PG Eriksson N Furlotte NA Verhoeven VJM. Genome-wide association Meta-analysis highlights light-induced signaling as a driver for refractive error. Nat Genet. (2018) 50:834–48. doi: 10.1038/s41588-018-0127-7, PMID: 29808027 PMC5980758

[130] Tedja MS Haarman AEG Meester-Smoor MA Kaprio J Mackey DA Guggenheim JA. Imi–myopia genetics report. Invest Ophthalmol Vis Sci. (2019) 60:M89–M105. doi: 10.1167/iovs.18-25965, PMID: 30817828 PMC6892384

[131] Verhoeven VJ Hysi PG Wojciechowski R Fan Q Kiefer AK Klaver CCW. Genome-wide mega-analysis on myopia and refractive error in Cream and 23andme. Invest Ophthalmol Vis Sci. (2014) 55:839.

[132] Verhoeven VJM Hysi PG Wojciechowski R Fan Q Guggenheim JA Höhn R. Genome-wide Meta-analyses of multiancestry cohorts identify multiple new susceptibility loci for refractive error and myopia. Nat Genet. (2013) 45:314–8. doi: 10.1038/ng.2554, PMID: 23396134 PMC3740568

[133] Kiefer AK Tung JY Do CB Hinds DA Mountain JL Francke U. Genome-wide analysis points to roles for extracellular matrix remodeling, the visual cycle, and neuronal development in myopia. PLoS Genet. (2013) 9:e1003299. doi: 10.1371/journal.pgen.1003299, PMID: 23468642 PMC3585144

[134] Haarman AEG Tedja MS Meester-Smoor MA Kaprio J Mackey DA Guggenheim JA. Consortium for refractive error and myopia (Cream): vision, Mission, and accomplishments In: Prakash G Iwata T, editors. Advances in Vision Research, Volume III: Genetic Eye Research Around the Globe. Singapore: Springer Singapore (2021). 381–407.

[135] Al-Shabrawey M Hussein K Wang F Wan M Elmasry K Elsherbiny N. Bone morphogenetic Protein-2 induces non-canonical inflammatory and oxidative pathways in human retinal endothelial cells. Front Immunol. (2021):11. doi: 10.3389/fimmu.2020.568795 PMC7878387 33584642

[136] Nasrabadi D Rezaeiani S Eslaminejad MB Shabani A. Improved protocol for Chondrogenic differentiation of bone marrow derived mesenchymal stem cells-effect of Pthrp and Fgf-2 on Tgfβ1/Bmp2-induced chondrocytes hypertrophy. Stem Cell Rev Rep. (2018) 14:755–66. doi: 10.1007/s12015-018-9816-y, PMID: 29691795

[137] Simões Sato AY Bub GL Campos AH. Bmp-2 and-4 produced by vascular smooth muscle cells from atherosclerotic lesions induce monocyte chemotaxis through direct Bmprii activation. Atherosclerosis. (2014) 235:45–55. doi: 10.1016/j.atherosclerosis.2014.03.030, PMID: 24814649

[138] Helbing T Rothweiler R Ketterer E Goetz L Heinke J Grundmann S. Bmp activity controlled by Bmper regulates the Proinflammatory phenotype of endothelium. Blood. (2011) 118:5040–9. doi: 10.1182/blood-2011-03-339762, PMID: 21900199

[139] Xu J Zhu D Sonoda S He S Spee C Ryan SJ. Over-expression of Bmp4 inhibits experimental choroidal neovascularization by modulating Vegf and Mmp-9. Angiogenesis. (2012) 15:213–27. doi: 10.1007/s10456-012-9254-4, PMID: 22392094 PMC3413482

[140] Siemerink MJ Hughes MR Dallinga MG Gora T Cait J Vogels IM. Cd34 promotes pathological epi-retinal neovascularization in a mouse model of oxygen-induced retinopathy. PLoS One. (2016) 11:e0157902. doi: 10.1371/journal.pone.0157902, PMID: 27352134 PMC4924789

[141] Zauhar R Biber J Jabri Y Kim M Hu J Kaplan L. As in real estate, location matters: cellular expression of complement varies between macular and peripheral regions of the retina and supporting tissues. Front Immunol. (2022):13. doi: 10.3389/fimmu.2022.895519 PMC9240314 35784369

[142] Kim C Smith KE Castillejos A Diaz-Aguilar D Saint-Geniez M Connor KM. The alternative complement pathway aids in vascular regression during the early stages of a murine model of proliferative retinopathy. FASEB J. (2016) 30:1300–5. doi: 10.1096/fj.15-280834 26631482 PMC4750413

[143] Suchting S Freitas C le Noble F Benedito R Bréant C Duarte A. The notch Ligand Delta-like 4 negatively regulates endothelial tip cell formation and vessel branching. Proc Natl Acad Sci U S A. (2007) 104:3225–30. doi: 10.1073/pnas.0611177104, PMID: 17296941 PMC1805603

[144] Lobov IB Renard RA Papadopoulos N Gale NW Thurston G Yancopoulos GD. Delta-like ligand 4 (Dll4) is induced by Vegf as a negative regulator of Angiogenic sprouting. Proc Natl Acad Sci U S A. (2007) 104:3219–24. doi: 10.1073/pnas.0611206104, PMID: 17296940 PMC1805530

[145] Kappas NC Zeng G Chappell JC Kearney JB Hazarika S Kallianos KG. The Vegf receptor Flt-1 spatially modulates Flk-1 signaling and blood vessel branching. J Cell Biol. (2008) 181:847–58. doi: 10.1083/jcb.200709114, PMID: 18504303 PMC2396811

[146] Chappell JC Cluceru JG Nesmith JE Mouillesseaux KP Bradley VB Hartland CM. Flt-1 (Vegfr-1) coordinates discrete stages of blood vessel formation. Cardiovasc Res. (2016) 111:84–93. doi: 10.1093/cvr/cvw091, PMID: 27142980 PMC4909163

[147] Nesmith JE Chappell JC Cluceru JG Bautch VL. Blood vessel anastomosis is spatially regulated by Flt1 during angiogenesis. Development. (2017) 144:889–96. doi: 10.1242/dev.145672, PMID: 28246215 PMC5374355

[148] Chappell JC Mouillesseaux KP Bautch VL. Flt-1 (vascular endothelial growth factor receptor-1) is essential for the vascular endothelial growth factor-notch feedback loop during angiogenesis. Arterioscler Thromb Vasc Biol. (2013) 33:1952–9. doi: 10.1161/ATVBAHA.113.301805, PMID: 23744993 PMC4521230

[149] Tosi GM Orlandini M Galvagni F. The controversial role of Tgf-Β in Neovascular age-related macular degeneration pathogenesis. Int J Mol Sci. (2018) 19:3363. doi: 10.3390/ijms19113363, PMID: 30373226 PMC6275040

[150] Zarkada G Howard JP Xiao X Park H Bizou M Leclerc S. Specialized endothelial tip cells guide Neuroretina vascularization and blood-retina-barrier formation. Dev Cell. (2021) 56:2237–2251.e6. doi: 10.1016/j.devcel.2021.06.021, PMID: 34273276 PMC9951594

[151] Goumans MJ Valdimarsdottir G Itoh S Rosendahl A Sideras P ten Dijke P. Balancing the activation state of the endothelium via two distinct Tgf-Beta type I receptors. EMBO J. (2002) 21:1743–53. doi: 10.1093/emboj/21.7.1743, PMID: 11927558 PMC125949

[152] Hu J Li T Du X Wu Q Le YZ. G protein-coupled receptor 91 signaling in diabetic retinopathy and hypoxic retinal diseases. Vis Res. (2017) 139:59–64. doi: 10.1016/j.visres.2017.05.001, PMID: 28539261 PMC5723215

[153] DeGraff AC Frieden J Ahlquist RP. Present state of alpha-and Beta-adrenergic drugs I. Adrenerg Recept Am Heart J. (1976) 92:661–4. doi: 10.1016/S0002-8703(76)80086-5, PMID: 10722

[154] Dal Monte M Martini D Latina V Pavan B Filippi L Bagnoli P. Beta-Adrenoreceptor Agonism influences retinal responses to hypoxia in a model of retinopathy of prematurity. Invest Ophthalmol Vis Sci. (2012) 53:2181–92. doi: 10.1167/iovs.11-9408, PMID: 22410551

[155] Lindgren I Altimiras J. Chronic prenatal hypoxia sensitizes Beta-adrenoceptors in the embryonic heart but causes postnatal desensitization. Am J Physiol Regul Integr Comp Physiol. (2009) 297:R258–64. doi: 10.1152/ajpregu.00167.2009, PMID: 19458283

[156] Casini G Dal Monte M Fornaciari I Filippi L Bagnoli P. The Β-adrenergic system as a possible new target for pharmacologic treatment of Neovascular retinal diseases. Prog Retin Eye Res. (2014) 42:103–29. doi: 10.1016/j.preteyeres.2014.06.001, PMID: 24933041

[157] Martinez-Camarillo JC Spee CK Trujillo-Sanchez GP Rodriguez A Hinton DR Giarola A. Blocking ocular sympathetic activity inhibits choroidal neovascularization. Front Neurosci. (2021) 15:780841. doi: 10.3389/fnins.2021.780841, PMID: 35082594 PMC8784868

[158] Steinle JJ. Review: role of camp signaling in diabetic retinopathy. Mol Vis. (2020) 26:355–8. PMID: 32476815 PMC7245604

[159] Biesecker KR Srienc AI Shimoda AM Agarwal A Bergles DE Kofuji P. Glial cell calcium signaling mediates capillary regulation of blood flow in the retina. J Neurosci. (2016) 36:9435–45. doi: 10.1523/JNEUROSCI.1782-16.2016, PMID: 27605617 PMC5013190

[160] Newman EA. Functional hyperemia and mechanisms of neurovascular coupling in the retinal vasculature. J Cereb Blood Flow Metab. (2013) 33:1685–95. doi: 10.1038/jcbfm.2013.145, PMID: 23963372 PMC3824187

[161] Novak J Parry LJ Matthews JE Kerchner LJ Indovina K Hanley-Yanez K. Evidence for local Relaxin ligand-receptor expression and function in arteries. FASEB J. (2006) 20:2352–62. doi: 10.1096/fj.06-6263com 17077312

[162] Jelinic M Leo CH Post Uiterweer ED Sandow SL Gooi JH Wlodek ME. Localization of Relaxin receptors in arteries and veins, and region-specific increases in compliance and bradykinin-mediated relaxation after in vivo Serelaxin treatment. FASEB J. (2014) 28:275–87. doi: 10.1096/fj.13-233429 24036884 PMC3868828

[163] Ng HH Jelinic M Parry LJ Leo CH. Increased superoxide production and altered nitric oxide-mediated relaxation in the aorta of Young but not old male Relaxin-deficient mice. Am J Phys Heart Circ Phys. (2015) 309:H285–96. doi: 10.1152/ajpheart.00786.2014, PMID: 25957220

[164] Madaan A Chaudhari P Nadeau-Vallée M Hamel D Zhu T Mitchell G. Müller cell-localized G-protein-coupled receptor 81 (Hydroxycarboxylic acid receptor 1) regulates inner retinal vasculature via Norrin/Wnt pathways. Am J Pathol. (2019) 189:1878–96. doi: 10.1016/j.ajpath.2019.05.016, PMID: 31220454

[165] Ando A Yang A Mori K Yamada H Yamada E Takahashi K. Nitric oxide is proangiogenic in the retina and choroid*. J Cell Physiol. (2002) 191:116–24. doi: 10.1002/jcp.10083, PMID: 11920687

[166] Lin C Toychiev A Ablordeppey R Slavi N Srinivas M Benavente-Perez A. Myopia alters the structural Organization of the Retinal Vasculature, Gfap-positive glia and ganglion cell layer thickness. Int J Mol Sci. (2022) 23:6202. doi: 10.3390/ijms23116202, PMID: 35682880 PMC9181442

[167] Haarman AEG Enthoven CA Tideman JWL Tedja MS Verhoeven VJM Klaver CCW. The complications of myopia: a review and Meta-analysis. Invest Ophthalmol Vis Sci. (2020) 61:49. doi: 10.1167/iovs.61.4.49 PMC7401976 32347918

[168] Sun J Wang Y Wang J. Choroidal arterial watershed zone topography and its relationship with maculopathy in highly myopic eyes. Eye. (2021) 35:2624–30. doi: 10.1038/s41433-021-01427-y, PMID: 33564136 PMC8376910

[169] Wong CW Phua V Lee SY Wong TY Cheung CMG. Is choroidal or scleral thickness related to myopic macular degeneration? Invest Ophthalmol Vis Sci. (2017) 58:907–13. doi: 10.1167/iovs.16-20742, PMID: 28166316

[170] Hsia Y Wang S-W Huang C-J Hung K-C Chen M-S Ho T-C. Clinical characteristics of highly myopic patients with asymmetric myopic atrophic maculopathy–analysis using multimodal imaging. Invest Ophthalmol Vis Sci. (2021) 62:21. doi: 10.1167/iovs.62.3.21, PMID: 33724293 PMC7980047

[171] Li J Zhou H Feinstein M Wong J Wang RK Chan L. Choriocapillaris changes in myopic macular degeneration. Transl Vis Sci Technol. (2022) 11:37. doi: 10.1167/tvst.11.2.37, PMID: 35201337 PMC8883151

[172] Ohno-Matsui K Lai TY Lai CC Cheung CM. Updates of pathologic myopia. Prog Retin Eye Res. (2016) 52:156–87. doi: 10.1016/j.preteyeres.2015.12.001 26769165

[173] Weinreb RN Aung T Medeiros FA. The pathophysiology and treatment of Glaucoma: a review. JAMA. (2014) 311:1901–11. doi: 10.1001/jama.2014.3192, PMID: 24825645 PMC4523637

[174] Chan KKW Tang F Tham CCY Young AL Cheung CY. Retinal vasculature in Glaucoma: a review. BMJ Open Ophthalmol. (2017) 1:e000032. doi: 10.1136/bmjophth-2016-000032, PMID: 29354699 PMC5721639

[175] Mitchell P Hourihan F Sandbach J Wang JJ. The relationship between Glaucoma and myopia - the Blue Mountains eye study. Ophthalmology. (1999) 106:2010–5. doi: 10.1016/S0161-6420(99)90416-5, PMID: 10519600

[176] Cedrone C Mancino R Ricci F Cerulli A Culasso F Nucci C. The 12-year incidence of Glaucoma and Glaucoma-related visual field loss in Italy: the Ponza eye study. J Glaucoma. (2012) 21:1–6. doi: 10.1097/IJG.0b013e3182027796, PMID: 21173704

[177] Czudowska MA Ramdas WD Wolfs RCW Hofman A De Jong PTVM Vingerling JR. Incidence of glaucomatous visual field loss: a ten-year follow-up from the Rotterdam study. Ophthalmology. (2010) 117:1705–12. doi: 10.1016/j.ophtha.2010.01.034, PMID: 20591487

[178] Kuzin AA Varma R Reddy HS Torres M Azen SP Los Angeles Latino Eye Study G. Ocular biometry and open-angle Glaucoma: the Los Angeles Latino eye study. Ophthalmology. (2010) 117:1713–9. doi: 10.1016/j.ophtha.2010.01.035, PMID: 20570359 PMC2934756

[179] Marcus MW de Vries MM Montolio FGJ Jansonius NM. Myopia as a risk factor for open-angle Glaucoma: a systematic review and Meta-analysis. Ophthalmology. (2011) 118:1989–94.e2. doi: 10.1016/j.ophtha.2011.03.012, PMID: 21684603

[180] Xu L Wang Y Wang S Wang Y Jonas JB. High myopia and Glaucoma susceptibility: the Beijing eye study. Ophthalmology. (2007) 114:216–20. doi: 10.1016/j.ophtha.2006.06.050, PMID: 17123613

[181] Memarzadeh F Ying-Lai M Chung J Azen SP Varma R. Blood pressure, perfusion pressure, and open-angle Glaucoma: the Los Angeles Latino eye study. Invest Ophthalmol Vis Sci. (2010) 51:2872–7. doi: 10.1167/iovs.08-2956, PMID: 20089880 PMC2891455

[182] Leske MC Wu SY Hennis A Honkanen R Nemesure B. Risk factors for incident open-angle Glaucoma: the Barbados eye studies. Ophthalmology. (2008) 115:85–93. doi: 10.1016/j.ophtha.2007.03.017, PMID: 17629563

[183] Fuchsjäger-Mayrl G Wally B Georgopoulos M Rainer G Kircher K Buehl W. Ocular blood flow and systemic blood pressure in patients with primary open-angle Glaucoma and ocular hypertension. Invest Ophthalmol Vis Sci. (2004) 45:834–9. doi: 10.1167/iovs.03-0461, PMID: 14985298

[184] Suwan Y Fard MA Geyman LS Tantraworasin A Chui TY Rosen RB. Association of Myopia with Peripapillary perfused capillary density in patients with Glaucoma: an optical coherence tomography angiography study. JAMA Ophthalmol. (2018) 136:507–13. doi: 10.1001/jamaophthalmol.2018.0776, PMID: 29621390 PMC6145659

[185] Mammo Z Heisler M Balaratnasingam C Lee S Yu DY Mackenzie P. Quantitative optical coherence tomography angiography of radial Peripapillary capillaries in Glaucoma, Glaucoma suspect, and Normal eyes. Am J Ophthalmol. (2016) 170:41–9. doi: 10.1016/j.ajo.2016.07.015, PMID: 27470061

[186] Ye J Lin J Shen M Chen W Zhang R Lu F. Reduced radial Peripapillary capillary in pathological myopia is correlated with visual acuity. Front Neurosci. (2022) 16:818530. doi: 10.3389/fnins.2022.818530, PMID: 35464317 PMC9033284

[187] Yamazaki Y Drance SM. The relationship between progression of visual field defects and retrobulbar circulation in patients with Glaucoma. Am J Ophthalmol. (1997) 124:287–95. doi: 10.1016/S0002-9394(14)70820-7, PMID: 9439354

[188] Findl O Rainer G Dallinger S Dorner G Polak K Kiss B. Assessment of optic disk blood flow in patients with open-angle Glaucoma. Am J Ophthalmol. (2000) 130:589–96. doi: 10.1016/S0002-9394(00)00636-X, PMID: 11078837

[189] Venkataraman ST Flanagan JG Hudson C. Vascular reactivity of optic nerve head and retinal blood vessels in glaucoma – a review. Microcirculation. (2010) 17:568–81. doi: 10.1111/j.1549-8719.2010.00045.x 21040122

[190] Németh J Michelson G Harazny J. Retinal microcirculation correlates with Ocular Wall thickness, axial eye length, and refraction in Glaucoma patients. J Glaucoma. (2001) 10:390–5. doi: 10.1097/00061198-200110000-00005, PMID: 11711836

[191] Samra WA Pournaras C Riva C Emarah M. Choroidal hemodynamic in myopic patients with and without primary open-angle Glaucoma. Acta Ophthalmol. (2013) 91:371–5. doi: 10.1111/j.1755-3768.2012.02386.x, PMID: 22458651

[192] Lin F Li F Gao K He W Zeng J Chen Y. Longitudinal changes in macular optical coherence tomography angiography metrics in primary open-angle Glaucoma with high myopia: a prospective study. Invest Ophthalmol Vis Sci. (2021) 62:30. doi: 10.1167/iovs.62.1.30, PMID: 33507229 PMC7846949

[193] Metelitsina TI Grunwald JE DuPont JC Ying GS Brucker AJ Dunaief JL. Foveolar choroidal circulation and choroidal neovascularization in age-related macular degeneration. Invest Ophthalmol Vis Sci. (2008) 49:358–63. doi: 10.1167/iovs.07-0526, PMID: 18172113 PMC3077130

[194] Wakabayashi T Ikuno Y Oshima Y Hamasaki T Nishida K. Aqueous concentrations of vascular endothelial growth factor in eyes with high myopia with and without choroidal neovascularization. J Ophthalmol. (2013) 2013:257381. doi: 10.1155/2013/257381, PMID: 23533702 PMC3606763

[195] Feng B Su W Chen Q Gan R Wang M Wang J. Quantitative analysis of retinal vasculature in rhegmatogenous retinal detachment based on ultra-Widefield fundus imaging. Front Med. (2021) 8:797479. doi: 10.3389/fmed.2021.797479, PMID: 35118092 PMC8804160

[196] Tolentino FI Lapus JV Novalis G Trempe CL Gutow GS Ahmad A. Fluorescein angiography of degenerative lesions of the peripheral fundus and Rhegmatogenous retinal detachment. Int Ophthalmol Clin. (1976) 16:13–29. doi: 10.1097/00004397-197601610-00005 931673

[197] Cardillo PF. Vascular changes in Rhegmatogenous retinal detachment. Ophthalmologica. (1983) 186:17–24. doi: 10.1159/000309255 6823410

[198] Ohno-Matsui K. Proposed classification of posterior staphylomas based on analyses of eye shape by three-dimensional magnetic resonance imaging and wide-field fundus imaging. Ophthalmology. (2014) 121:1798–809. doi: 10.1016/j.ophtha.2014.03.035, PMID: 24813630

[199] Nie F Zhang L Cao M Zhou D Liu K Ouyang J. Impact of Peripapillary staphylomas on the vascular and structural characteristics in myopic eyes: a propensity score matching analysis. Graefes Arch Clin Exp Ophthalmol. (2023) 2013:257381. doi: 10.1007/s00417-022-05966-2, PMID: 36617582 PMC10271889

[200] Nie F Ouyang J Tang W Luo L Cao M Zhang L. Posterior staphyloma is associated with the microvasculature and microstructure of myopic eyes. Graefes Arch Clin Exp Ophthalmol. (2021) 259:2119–30. doi: 10.1007/s00417-020-05057-0, PMID: 33404680 PMC8352845

[201] Klein BE Klein R Jensen SC Linton KL. Hypertension and Lens opacities from the beaver dam eye study. Am J Ophthalmol. (1995) 119:640–6. doi: 10.1016/S0002-9394(14)70223-5, PMID: 7733190

[202] Klein BE Klein R Lee KE. Diabetes, cardiovascular disease, selected cardiovascular disease risk factors, and the 5-year incidence of age-related cataract and progression of Lens opacities: the beaver dam eye study. Am J Ophthalmol. (1998) 126:782–90. doi: 10.1016/S0002-9394(98)00280-3, PMID: 9860001

[203] Grieshaber MC Koçak I Dubler B Flammer J Orgül S. Retrobulbar blood flow in patients with cataract. Br J Ophthalmol. (2006) 90:1512–5. doi: 10.1136/bjo.2006.101261, PMID: 16885186 PMC1857537

[204] Jakobsson L Bentley K Gerhardt H. Vegfrs and notch: a dynamic collaboration in vascular patterning. Biochem Soc Trans. (2009) 37:1233–6. doi: 10.1042/BST0371233, PMID: 19909253

